# Supplementary material for: An entosis-like process induces mitotic disruption in Pals1 microcephaly pathogenesis
Source: Nat Commun. 2023 Jan 5;14:82. doi: 10.1038/s41467-022-35719-y (PMC9816111; doi:10.1038/s41467-022-35719-y)
Supplement: Supplementary file 1 — Supplementary Information [file 41467_2022_35719_MOESM1_ESM.pdf]

Supplementary Information
An entosis-like process induces mitotic disruption in Pals1 microcephaly pathogenesis

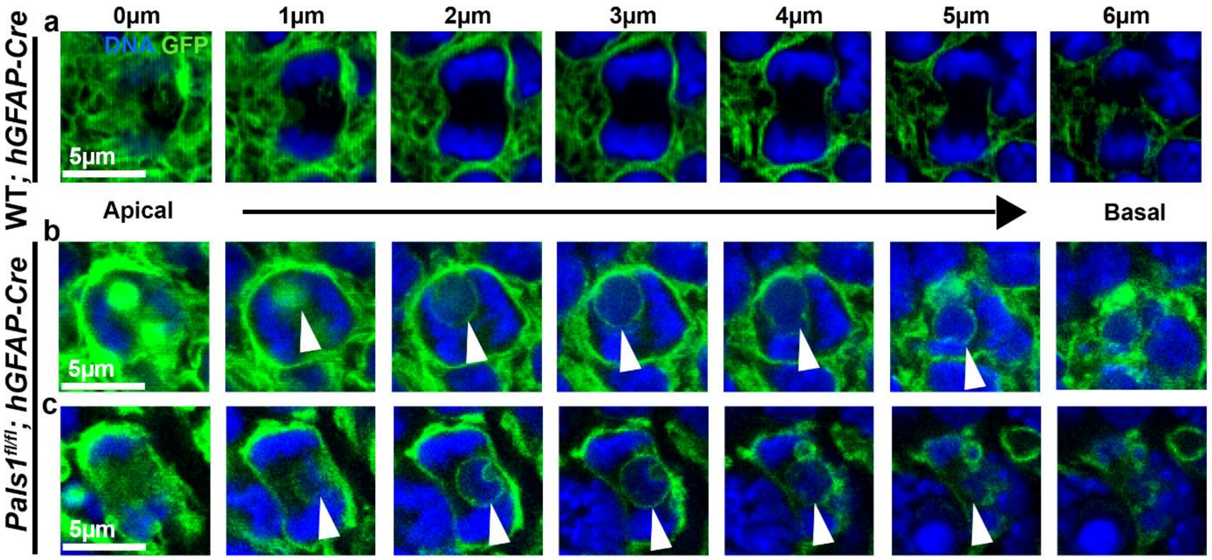

Supplementary Figure. 1. PALS1-deficient neural progenitors display cell-in-cell structures. (a) Representative images of WT cells imaged in z-stacks taken through dividing neural progenitors at the apical surface of the cortex. Cell membranes are green, and cellular DNA is blue. Images are from experiments including WT n=3 brains, 20 cells per brain. **(b-c)** Representative images of PALS-deficient neural progenitors imaged in z-stacks at the apical surface of the cortex. Images are from experiments including *Pals1*$^{fl/fl}$*; hGFAP-Cre* n=4 brains, 20 cells per brain. Each cell is in cytokinesis as denoted by elongated green membrane surrounding two separate sets of chromosomes. White arrows indicate cell-in-cell structures present in *Pals1*$^{fl/fl}$*; hGFAP-Cre* cells. Example cells are those presented in Figure 1b. Scale bars: 5 µm. Source data are provided as a Source Data file.

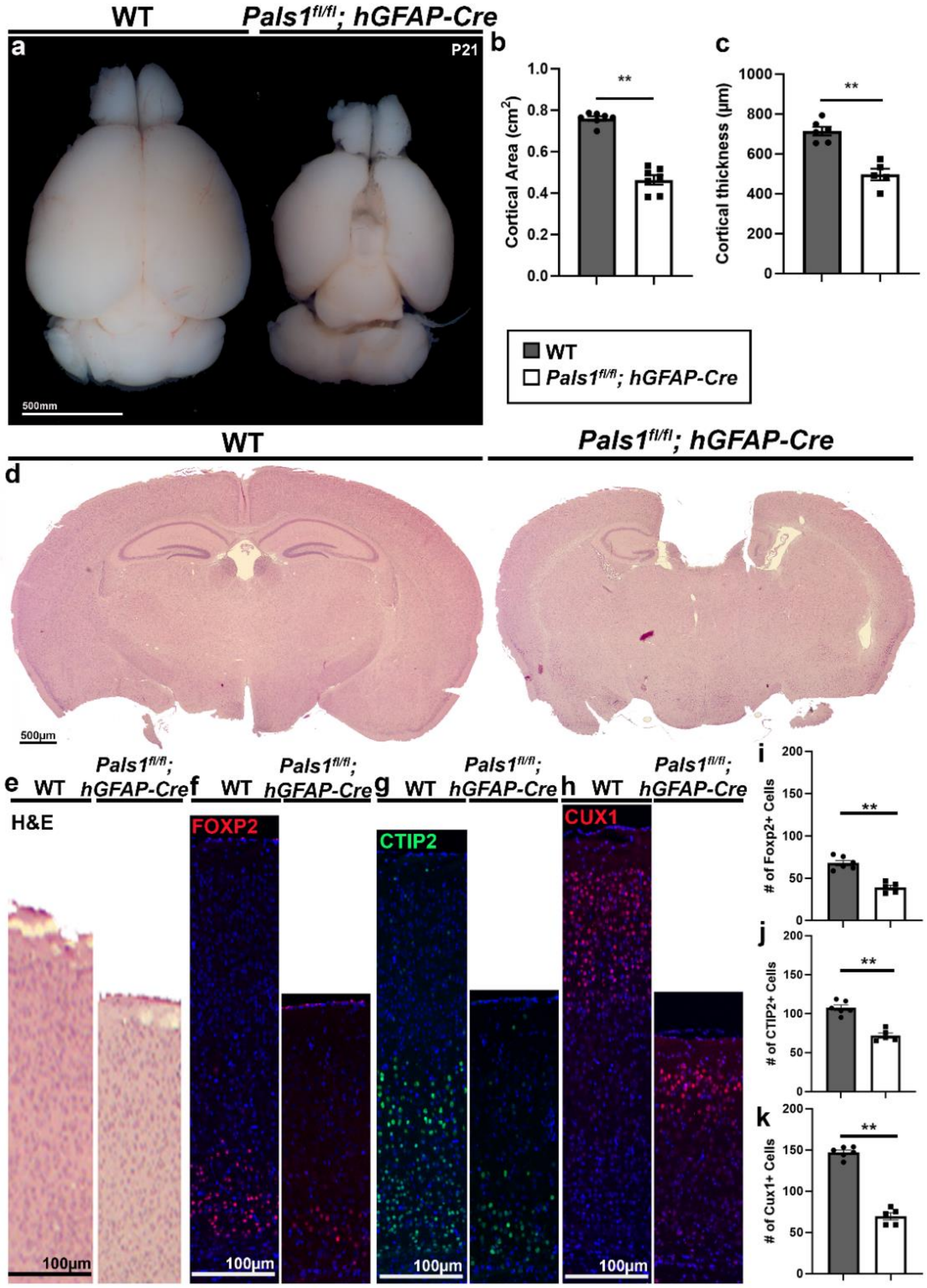

Supplementary Figure. 2. *Pals1* deletion results in small cortices and reduced neuron numbers. (a-d) Representative images of whole brains and histology of cortices at P21. Quantification of cortical surface area and thickness (WT n=6 brains, *Pals1$^{fl/fl}$; hGFAP-Cre* n=5 brains, surface area P=<.0001, thickness P=.0002). **(e-k)** Analysis and quantification of cortical cell populations at P21 including early-born (FOXP2^{+}, red and CTIP2^{+}, green) and late-born neurons (CUX1^{+}, red) (WT n=6 brains, *Pals1$^{fl/fl}$; hGFAP-Cre* n=5 brains, P=<.0001). Data for the graphs are presented as mean ± SEM, and statistical analysis was done using a two-tailed Student t-test. Scale bars: (a) 500 nm, (d) 500 µm, (e-h) 100 µm. Source data are provided as a Source Data file.

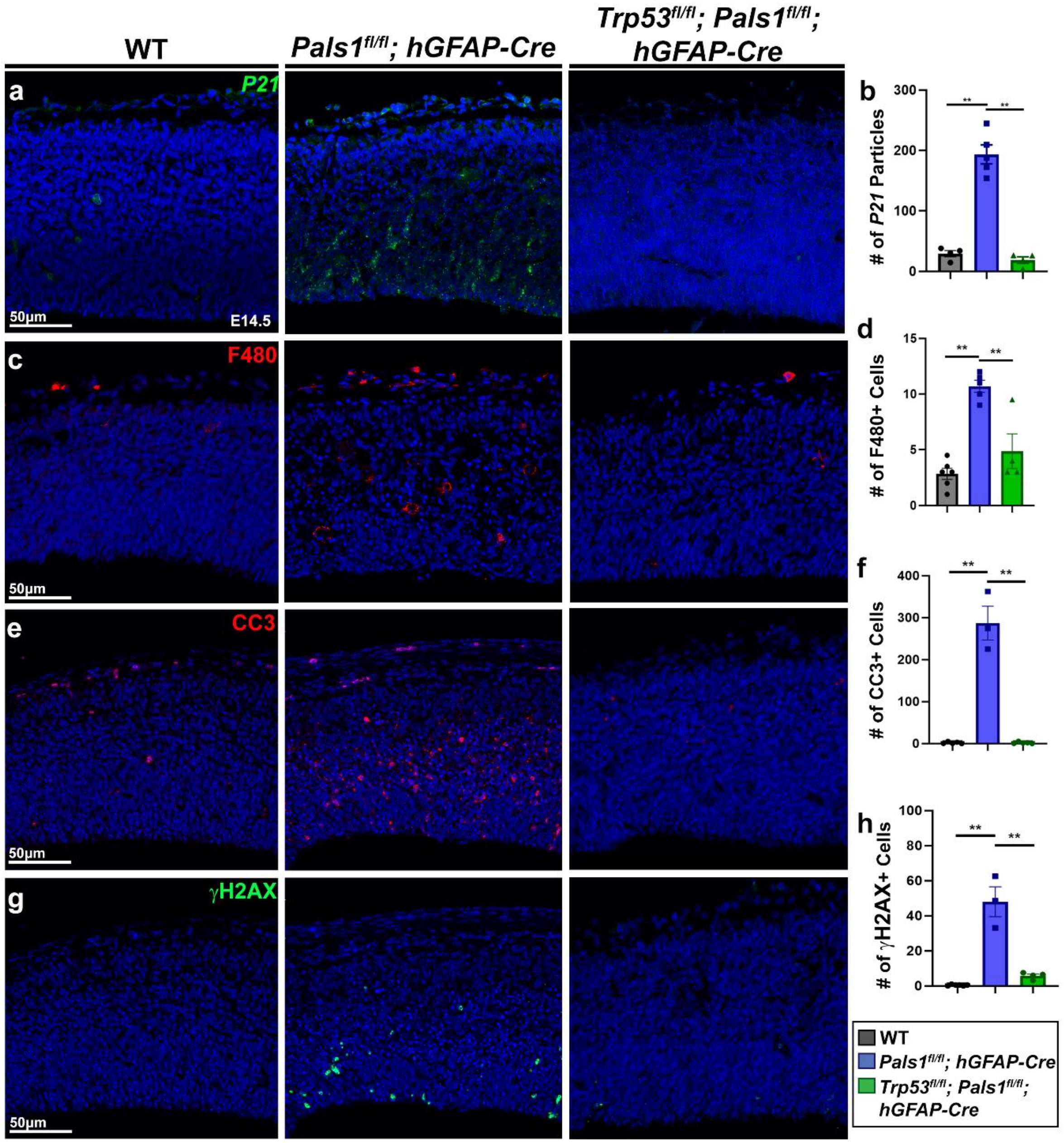

Supplementary Figure. 3. P53 activation mediates cortical cell death in *Pals1* mutants. (a-b) RNAScope and quantification for *P21* transcripts at E14.5 in green (WT n=4 brains, *Pals1$^{fl/fl}$; hGFAP-Cre* n=5 brains, *Trp53$^{fl/fl}$; Pals1$^{fl/fl}$; hGFAP-Cre* n=4 brains, P=<.0001, F=79.89, dof=12). **(c-d)** Representative images and quantification of activated microglia (F480^{+}, red) at E14.5 (WT n=6 brains, *Pals1$^{fl/fl}$; hGFAP-Cre* n=5 brains, *Trp53$^{fl/fl}$; Pals1$^{fl/fl}$; hGFAP-Cre* n=4 brains, P=<.0001, F=24.91, dof=14). **(e-h)** Representative images and quantification of apoptotic cells (CC3^{+}, red), and cells with DNA damage (γH2AX^{+}, green) at E14.5 (WT n=5 brains, *Pals1$^{fl/fl}$; hGFAP-Cre* n=3 brains, *Trp53$^{fl/fl}$; Pals1$^{fl/fl}$; hGFAP-Cre* n=4 brains, cell death (P=<.0001, F=96.34, dof=12), DNA damage (P=<.0001, F=54.03, dof=12). Data for the graphs are presented as mean ± SEM, and statistical analysis was done using a one-way ANOVA followed by a post-hoc Tukey test. Scale bars: 50 µm. Source data are provided as a Source Data file.

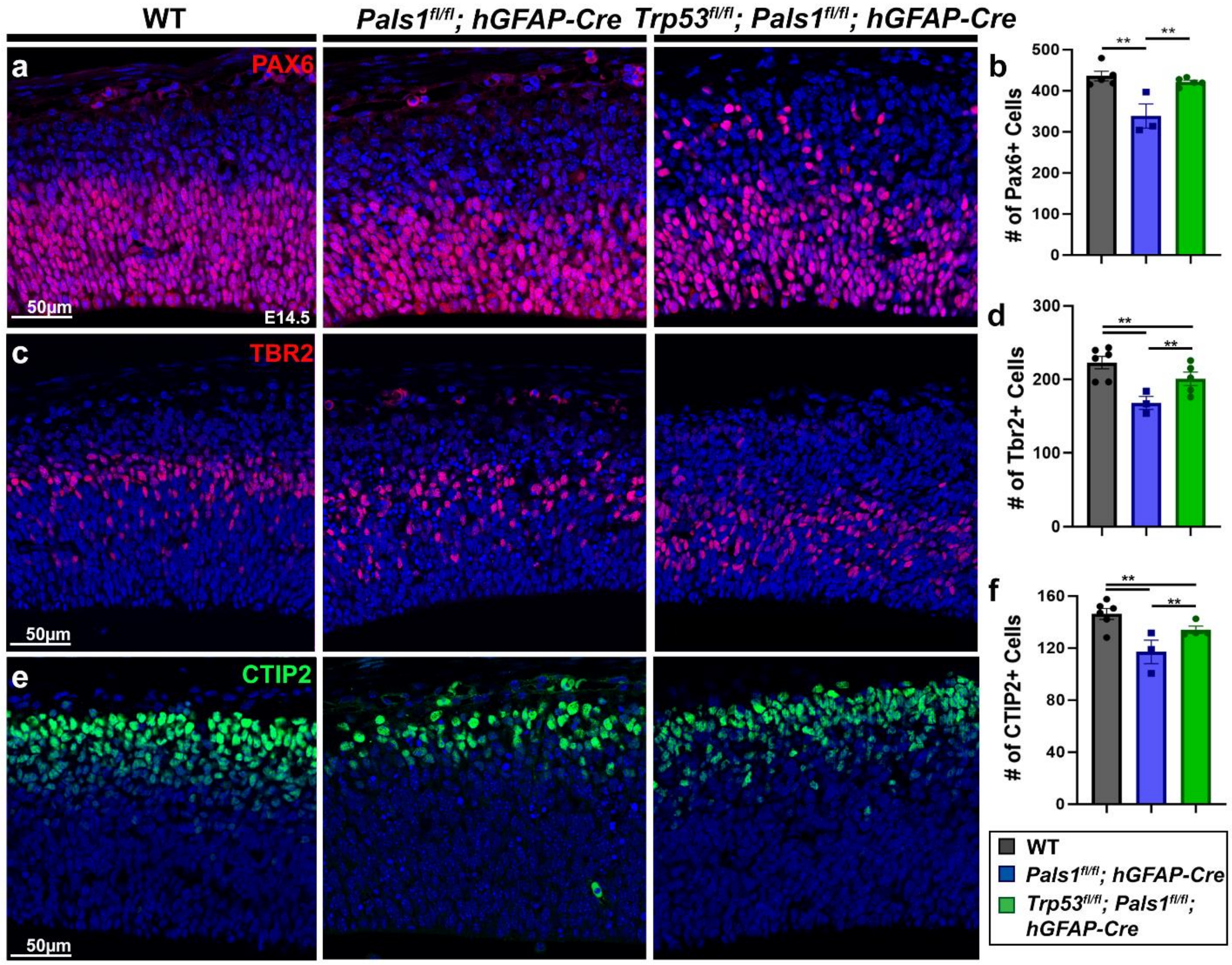

Supplementary Figure. 4. *Trp53* co-deletion significantly rescues cortical cell numbers at E14.5. (a-b) Immunostaining and quantification of $PAX6^+$ apical progenitors in red at E14.5 (WT n=5 brains, *Pals1*$^{fl/fl}$*; hGFAP-Cre* n=3 brains, *TrpP53*$^{fl/fl}$*; Pals1*$^{fl/fl}$*; hGFAP-Cre* n=5 brains, P=.002, F=11.47, dof=12). **(c-d)** Immunostaining and quantification of intermediate progenitors ($TBR2^+$, red) at E14.5 (WT n=6 brains, *Pals1*$^{fl/fl}$*; hGFAP-Cre* n=3 brains, *Trp53*$^{fl/fl}$*; Pals1*$^{fl/fl}$*; hGFAP-Cre* n=5 brains, P=.008, F=7.66, dof=13). **(e-f)** Immunostaining and quantification of early-born neurons ($CTIP2^+$, green) at E14.5 (WT n=6 brains, *Pals1*$^{fl/fl}$*; hGFAP-Cre* n=3 brains, *Trp*53$^{fl/fl}$; *Pals1*$^{fl/fl}$*; hGFAP-Cre* n=4 brains, P=.009, F=7.72, dof=12). Data for the graphs are presented as mean ± SEM, and statistical analysis was done using a one-way ANOVA followed by a post-hoc Tukey test. Scale bars: 50 µm. Source data are provided as a Source Data file.

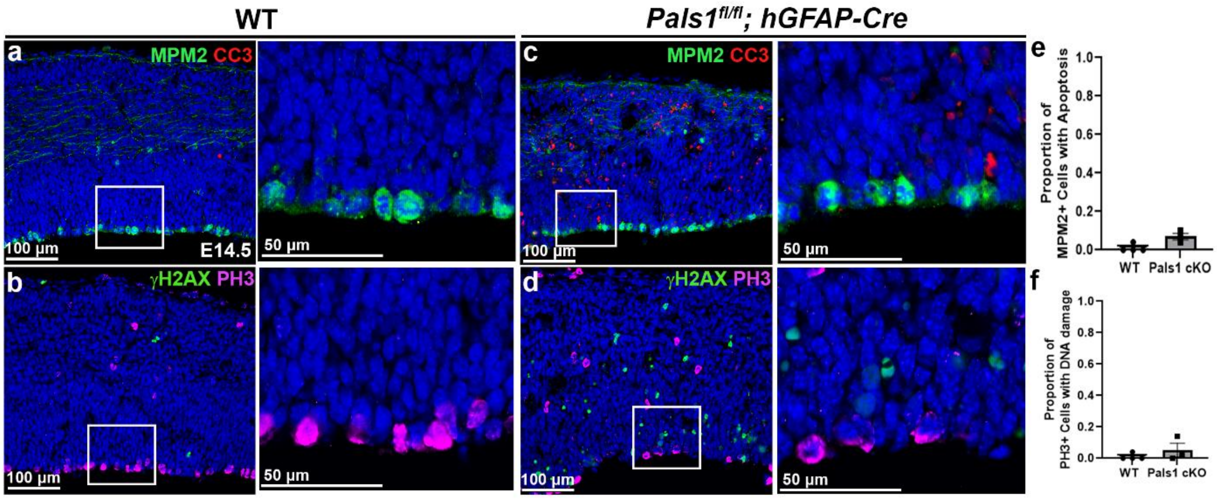

Supplementary Figure. 5. Dividing neural progenitors and CIC structures rarely undergo cell death in *Pals1* mutants. (a-d) Representative images of immunostaining for $MPM2^+$ (green) or $PH3^+$ (magenta) mitotic neural progenitors with markers for apoptotic cell death ($CC3^+$, red) or DNA damage ($\gamma H2AX^+$, green). **(e-f)** Quantification of the proportion of dividing cells ($MPM2^+$ or $PH3^+$) that are also undergoing apoptosis ($CC3^+$) or contain DNA damage ($\gamma H2AX^+$) at E14.5 (WT n=3 brains, *Pals1$^{fl/fl}$; hGFAP-Cre* n=3 brains, Cell death (P=.05), DNA damage (P=.43). Data for the graphs are presented as mean ± SEM, and statistical analysis was done using a two-tailed Student t-test. Scale bars: 100 µm, 50 µm. Source data are provided as a Source Data file.

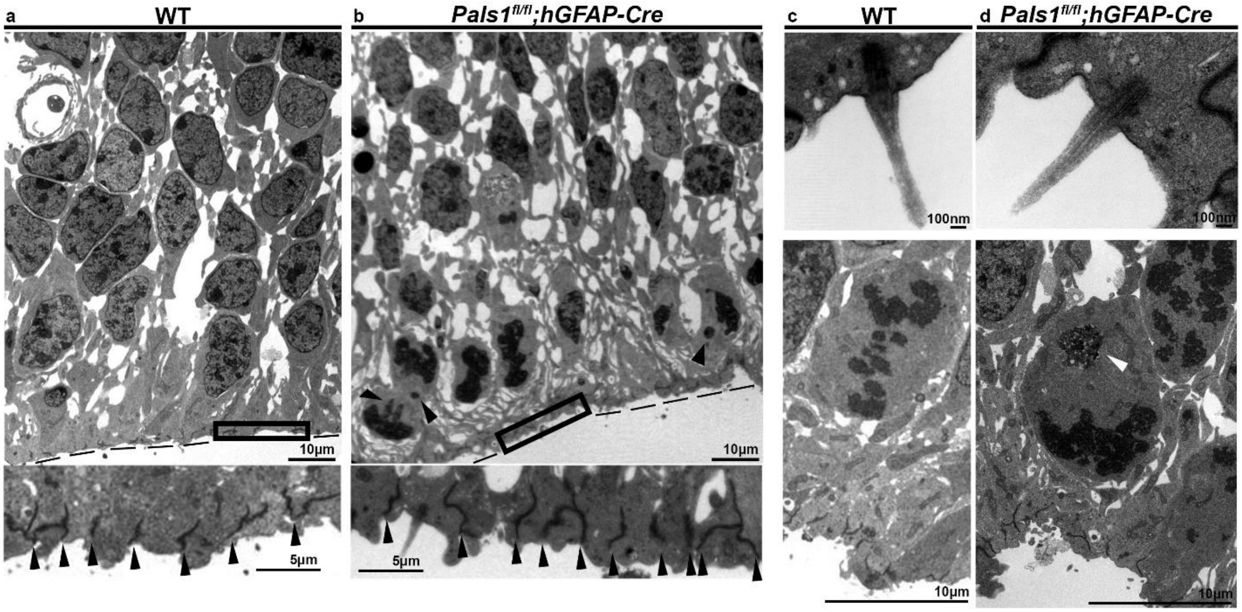

Supplementary Figure. 6. *Pals1* deletion causes mitotic alterations in cortical progenitors but does not affect adherens junctions. (a-b) Representative images from transmission electron microscopy done at E13.5 show dividing cells at the apical surface of the cortex and their cellular junctions. Black arrows indicate micronuclei and lagging chromosomes in *Pals1*$^{fl/fl}$*; hGFAP-Cre* cells. Dashed black lines indicate the apical surface of the cortex. Black boxes represent apical surface area enlarged in insets. Black arrows in inset images represent adherens junctions at the apical surface. **(c-d)** Representative images of cilia at E13.5 and examples of autophagic structures in *Pals1*$^{fl/fl}$*; hGFAP-Cre* cells resembling micronuclei as indicated by white arrow. For each of the features in this figure, 2 animals per group were examined. Scale bars: (a-b) 10 µm – 5 µm, (c-d) 100 nm – 10 µm. Source data are provided as a Source Data file.

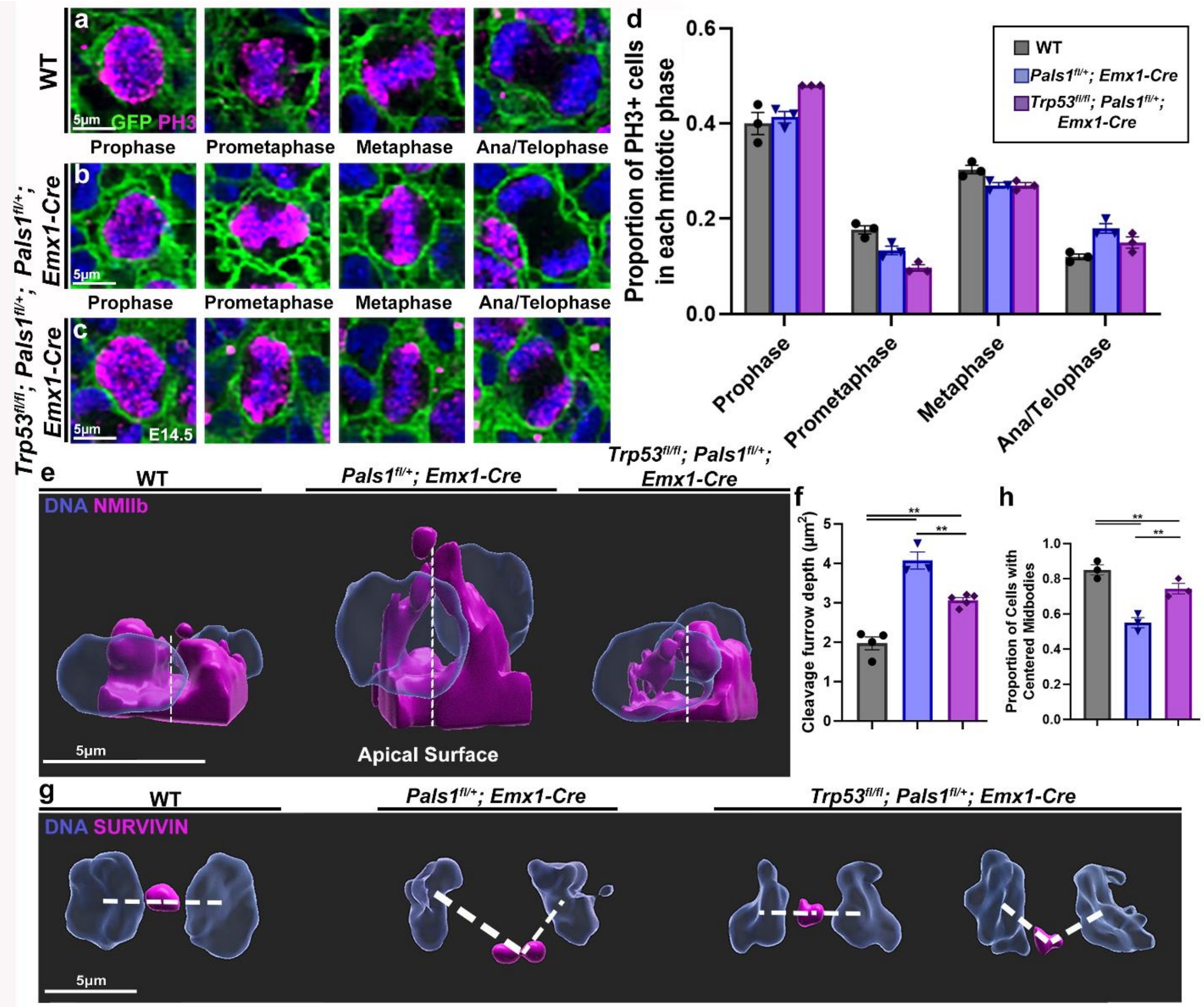

Supplementary Figure. 7. Mitotic defects caused by loss of PALS1 are significantly rescued by *Trp53* co-deletion. (a-d) Representative images at E14.5 of apical progenitors undergoing each stage of mitosis. Apical progenitors were visualized through apical explant staining for GFP in green and PH3 in magenta to amplify membrane-bound GFP and identify the mitotic stage. Quantification of the proportion of cells in each mitotic stage (WT n=3 brains, *Pals1*$^{fl/+}$*; Emx1-Cre* n=3 brains, *Trp53*$^{fl/fl}$*; Pals1*$^{fl/+}$*; Emx-1 Cre* n=3 brains). **(e-f)** IMARIS 3D models of cells and quantification from apical explants immunostained for NMIIB at E14.5 show cleavage furrow depth where NMIIb staining is magenta and DNA is blue. Dotted white lines indicate cleavage furrow depth measured and compared in F. (WT n=4 brains, *Pals1*$^{fl/+}$*; Emx1-Cre* n=3 brains, *Trp53*$^{fl/fl}$*; Pals1*$^{fl/+}$*; Emx1-Cre* n=5 brains, 5 cells per brain P=<.0001, F=29.81, dof=14). **(g-h)** IMARIS 3D models of SURVIVIN labeling of cell midbodies and quantification of the proportion of centered midbodies where SURVIVIN staining is magenta and DNA is blue. Dotted white lines indicate midbody placement with regards to chromosome position (WT n=3 brains, *Pals1*$^{fl/+}$*; Emx1-Cre* n=3 brains, *Trp53*$^{fl/fl}$*; Pals1*$^{fl/+}$*; Emx1-Cre* n=3 brains, 10 cells per brain P=.0006, F=18.32, dof=11). Data are presented as mean ± SEM, and statistical analysis for the data in these graphs was done using a one-way ANOVA followed by a post-hoc Tukey test. Scale bars: 5 µm. Source data are provided as a Source Data file.

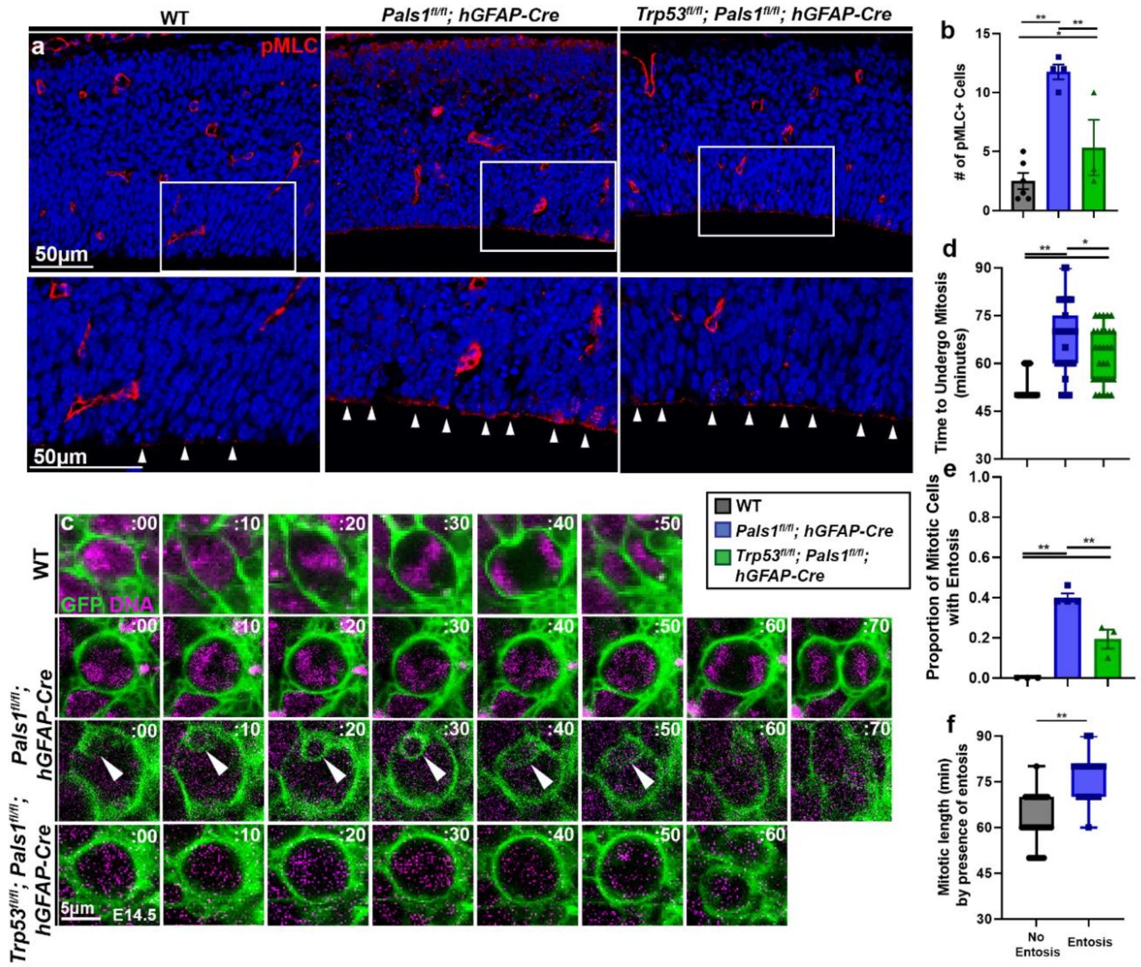

Supplementary Figure. 8. *Pals1* deletion results in mitotic defects that are significantly rescued by *Trp53* co-deletion. (a-b) Immunostaining and quantification of pMLC in red at E14.5. White boxes indicate areas of interest magnified below. White arrows indicate areas with pMLC expression. (WT n=6 brains, *Pals1*$^{fl/fl}$*; hGFAP-Cre* n=4 brains, *Trp53*$^{fl/fl}$*; Pals1*$^{fl/fl}$*; hGFAP-Cre* n=3 brains, P=.0007, F=16.67, dof=12). **(c-d)** Representative images of apical progenitors undergoing mitosis at E14.5 from both WT, *Pals1* cKO, and *Trp53* co-deletion animals (where cell membranes are green, and DNA is magenta) and quantification of the time to undergo mitosis. White arrows indicate CIC structures (WT n=3 brains, *Pals1*$^{fl/fl}$*; hGFAP-Cre* n=4 brains, *Trp53*$^{fl/fl}$*; Pals1*$^{fl/fl}$*; hGFAP-Cre* n=3 brains, 20 cells per brain, P=<.0001, F=28.42, dof=112). **(e)** Quantification of the proportion of mitotic cells displaying CIC structures (WT n=3 brains, *Pals1*$^{fl/fl}$*; hGFAP-Cre* n=4 brains, *Trp53*$^{fl/fl}$*; Pals1*$^{fl/fl}$*; hGFAP-Cre* n=3 brains, 20 cells per brain, P=<.0001, F=53.64, dof=9). (**f**) Quantification of the time to undergo mitosis by the presence of entosis in Pals1 cKO and Trp53 co-deletion from time-lapse imaging at E14.5 (No Entosis n=4 brains, Entosis n=4 brains, 20 cells per brain P=<.0001). For (c-f), data for WT and *Pals1* cKO groups reflects the same experiments performed for Figure 1 with the addition of new experiments for *Trp53* co-deletion. Data for the graphs are presented as mean ± SEM, and statistical analysis was done using a two-tailed student t-test, or a one-way ANOVA followed by a post-hoc Tukey test. Box plots: center line, median; box limits, upper and lower quartiles; whiskers, 1.5x interquartile range; points, all data represented. Scale bars: (a) 5 µm, (e) 50 µm. Source data are provided as a Source Data file.

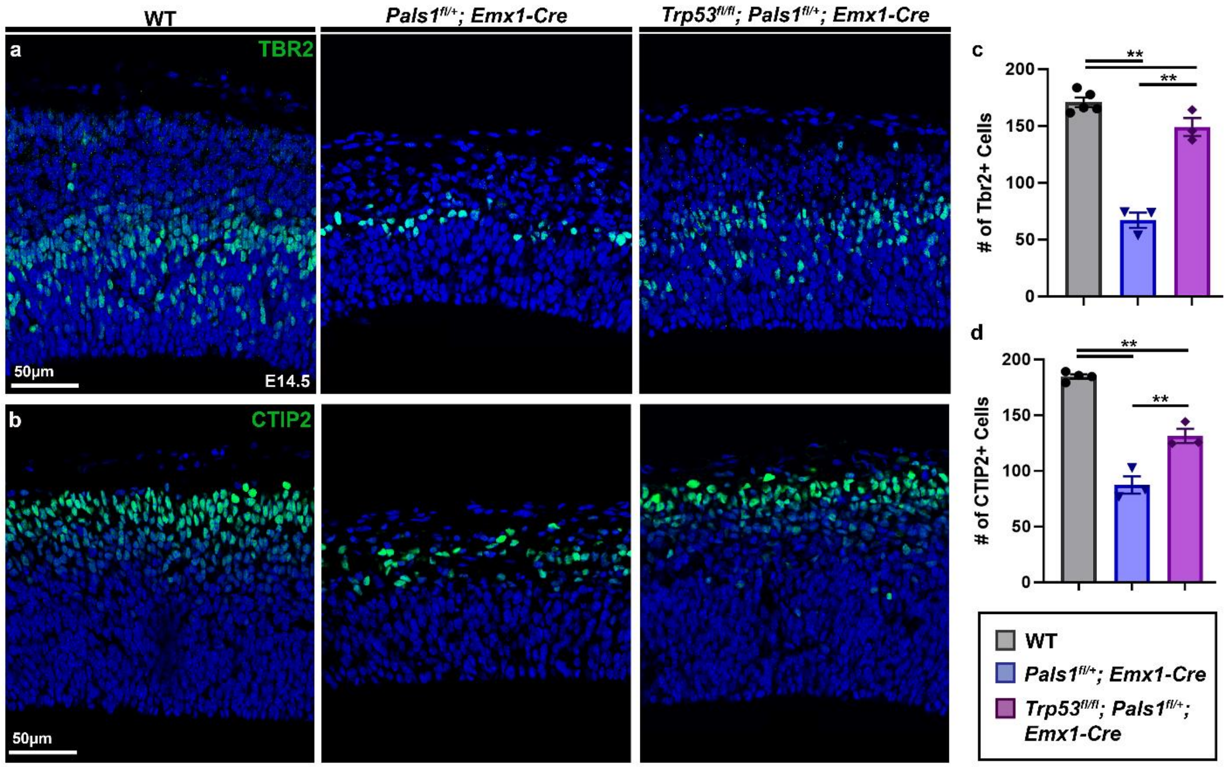

Supplementary Figure. 9. *Trp53* co-deletion significantly rescues cortical cell numbers at E14.5 in *Pals1* mutants. (a-d) Representative images and quantification of cortical labeling for intermediate progenitors (TBR2$^+$, green) and early-born neurons (CTIP2$^+$, green) at E14.5 (WT n=5 brains, *Pals1$^{fl/+}$; Emx1-Cre* n=3 brains, *Trp53$^{fl/fl}$; Pals1$^{fl/+}$; Emx1-Cre* n=3 brains, TBR2 (P=<.0001, F=38.69, dof=13), CTIP2 (P=<.0001, F=154.8, dof=13). Data for the graphs in this figure are presented as mean ± SEM, and statistical analysis was done using a one-way ANOVA followed by a post-hoc Tukey test. Scale bars: 50 μm. Source data are provided as a Source Data file.

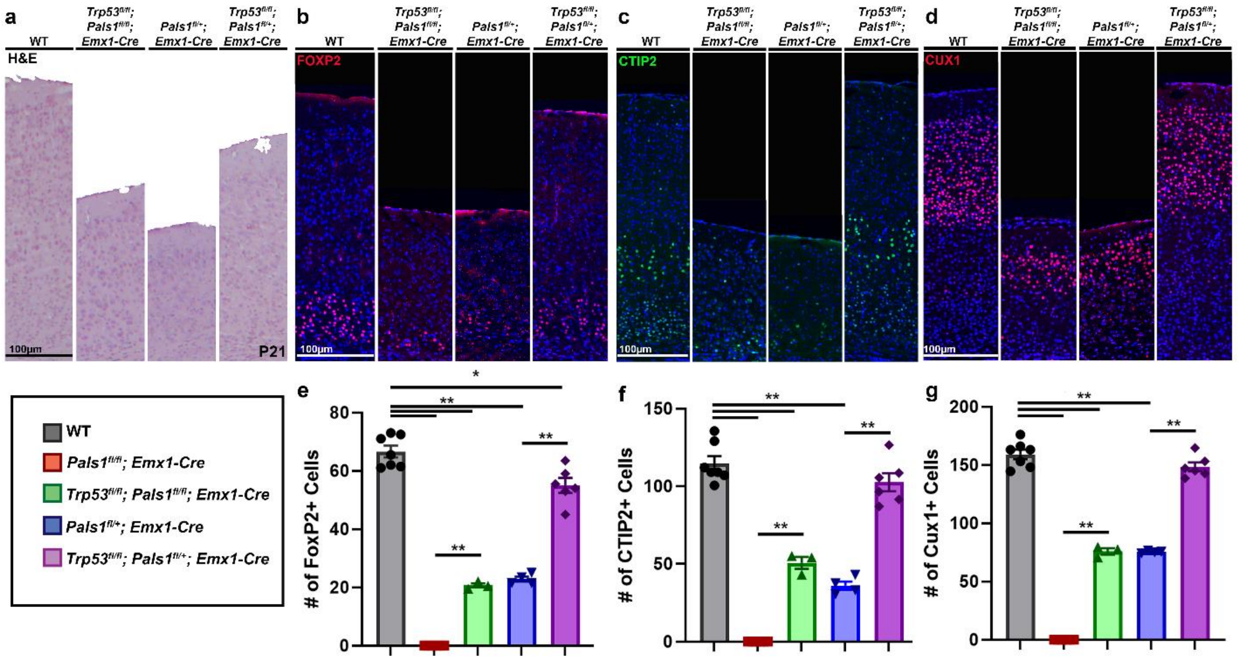

Supplementary Figure. 10. *Trp53* co-deletion significantly rescues cortical neuron numbers at P21. (a-d) Representative images of cortical labeling with H&E, FOXP2$^+$ (red) and CTIP2$^+$ (green) early-born neurons, and CUX1$^+$ (red) late-born neurons in WT, heterozygous *Pals1* deletion, and both homozygous and heterozygous double mutants at P21. Homozygous *Pals1* deletion results in no cortical development and is not pictured. **(e-g)** Quantification of neuronal cell numbers (WT n=7 brains, *Pals1$^{fl/fl}$; Emx1-Cre* n=6 brains, *Trp53$^{fl/fl}$; Pals1$^{fl/fl}$; Emx1-Cre* n=3 brains, *Pals1$^{fl/+}$; Emx1-Cre* n=4 brains, *Trp53$^{fl/fl}$; Pals1$^{fl/+}$; Emx1-Cre* n=6 brains, FOXP2 (P=<.0001, F=247.2, dof=25), CTIP2 (P=<.0001, F=133.2, dof=25), CUX1 (P=<.0001, F=444.1, dof=25). Data for the graphs are presented as mean ± SEM, and statistical analysis was done using a one-way ANOVA followed by a post-hoc Tukey test. Scale bars: 100 µm. Source data are provided as a Source Data file.

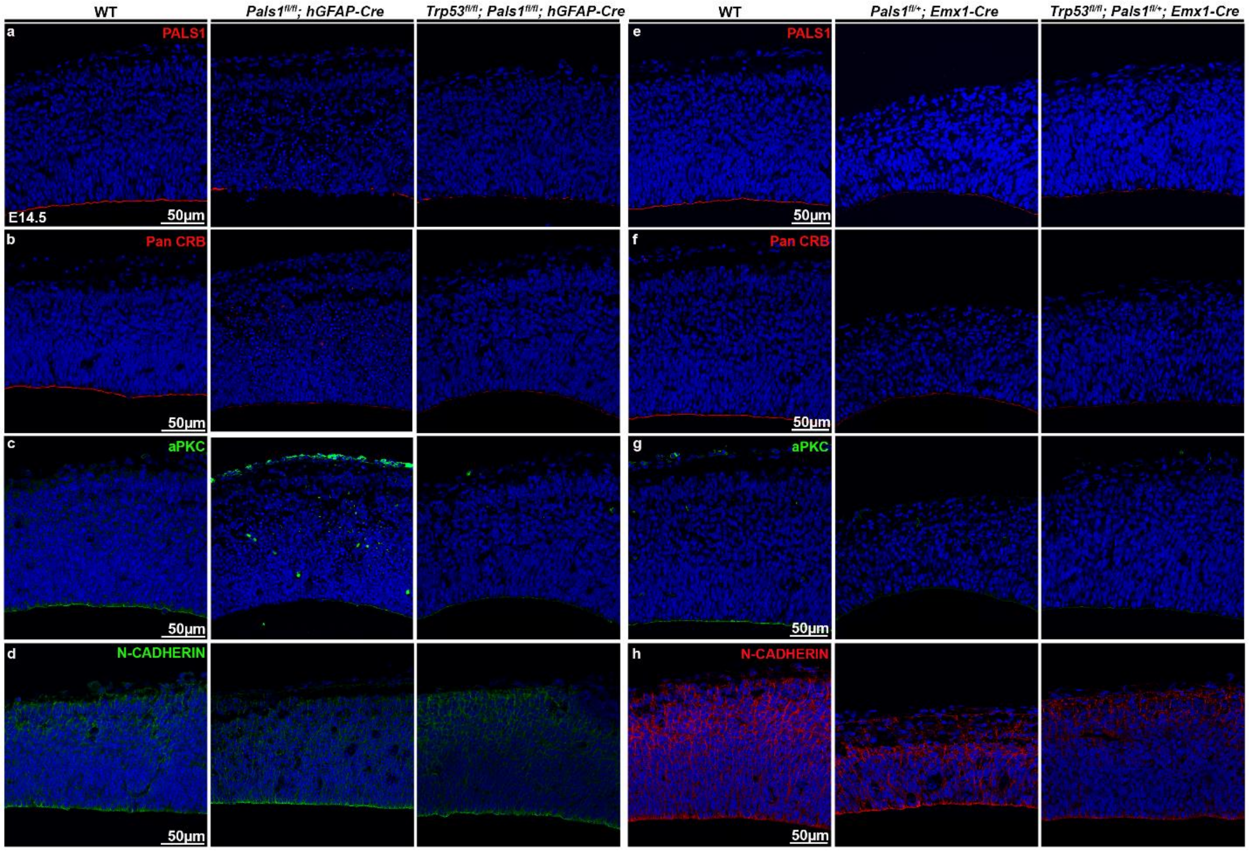

Supplementary Figure. 11. Loss of PALS1 causes apical polarity complex disruption regardless of *Trp53* deletion. (a-c) Representative images of immunostaining of apical polarity complex proteins PALS1 (red), Pan-CRB (red) and aPKC (green) at the cortical ventricular surface at E14.5 when *Pals1* or *Pals1* and *Trp53* are co-deleted using *hGFAP-Cre*. Due to PALS1 loss, apical polarity complexes are disrupted, as shown. **(d)** Immunostaining for adherens junction marker N-cadherin (green in d, red in h). **(e-h)** *Emx1-Cre; Pals1* heterozygotes display similar PALS1 reduction and apical complex disruption regardless of *Trp53* deletion. Adherens junctions remain unchanged by PALS1 loss at E14.5. For each of the immunostaining markers in this figure, 3 animals were examined per group. Scale bars: 50 µm. Source data are provided as a Source Data file.

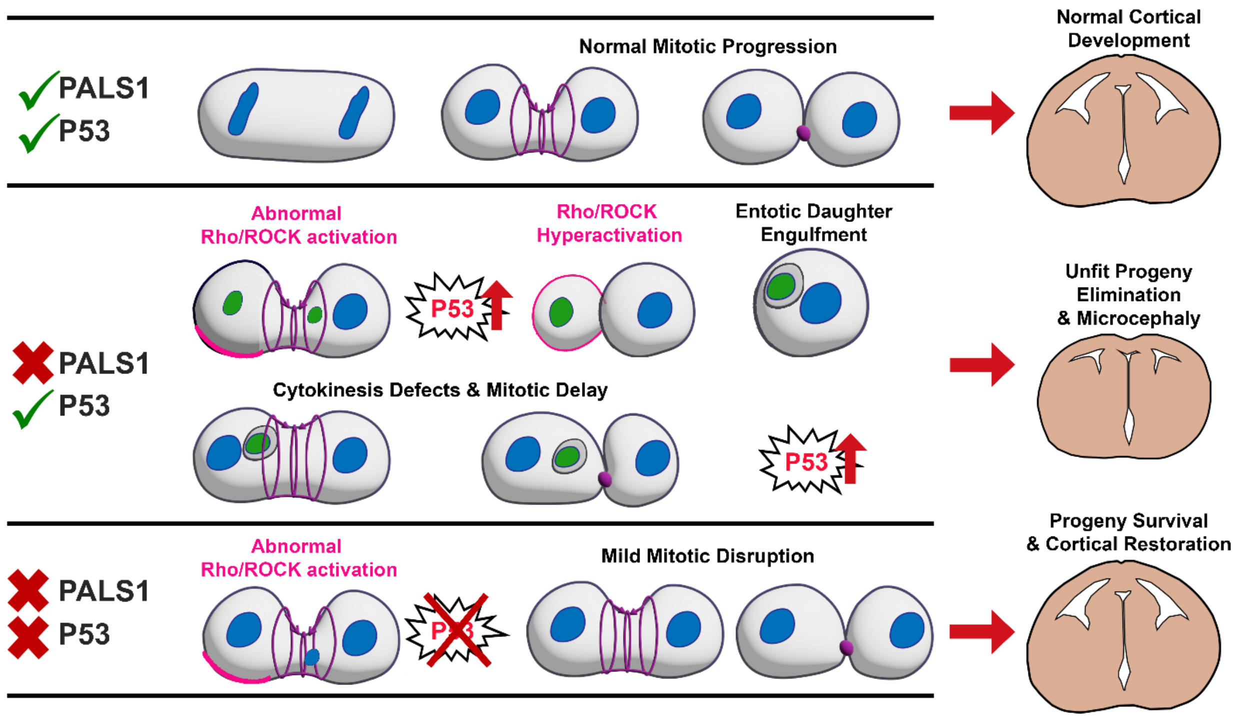

Supplementary Figure. 12. Proposed mechanism of cellular effects caused by *Pals1* deletion. PALS1 reduction causes mild lengthening of mitosis and chromosome abnormalities due to abnormal Rho-ROCK activation. These changes cause P53 activation that exacerbates Rho-ROCK hyperactivation and cause entosis. Increased mitosis length and chromosomal abnormalities such as micronuclei induce P53 activation. P53 activation further enhances Rho-ROCK activity and generation of CIC structures. CIC structures resulting from entosis result in further mitotic defects and reduced cellular fitness of neural progenitor progeny which may subsequently undergo cell death. When P53 is removed, hyperactivation of Rho-ROCK no longer occurs, and subsequent entosis and cytokinesis defects are significantly rescued, along with apoptotic cell death of progeny.
