# Supplementary figures and images for: An entosis-like process induces mitotic disruption in Pals1 microcephaly pathogenesis
Source: Nat Commun. 2023 Jan 5;14:82. doi: 10.1038/s41467-022-35719-y (PMC9816111; doi:10.1038/s41467-022-35719-y)

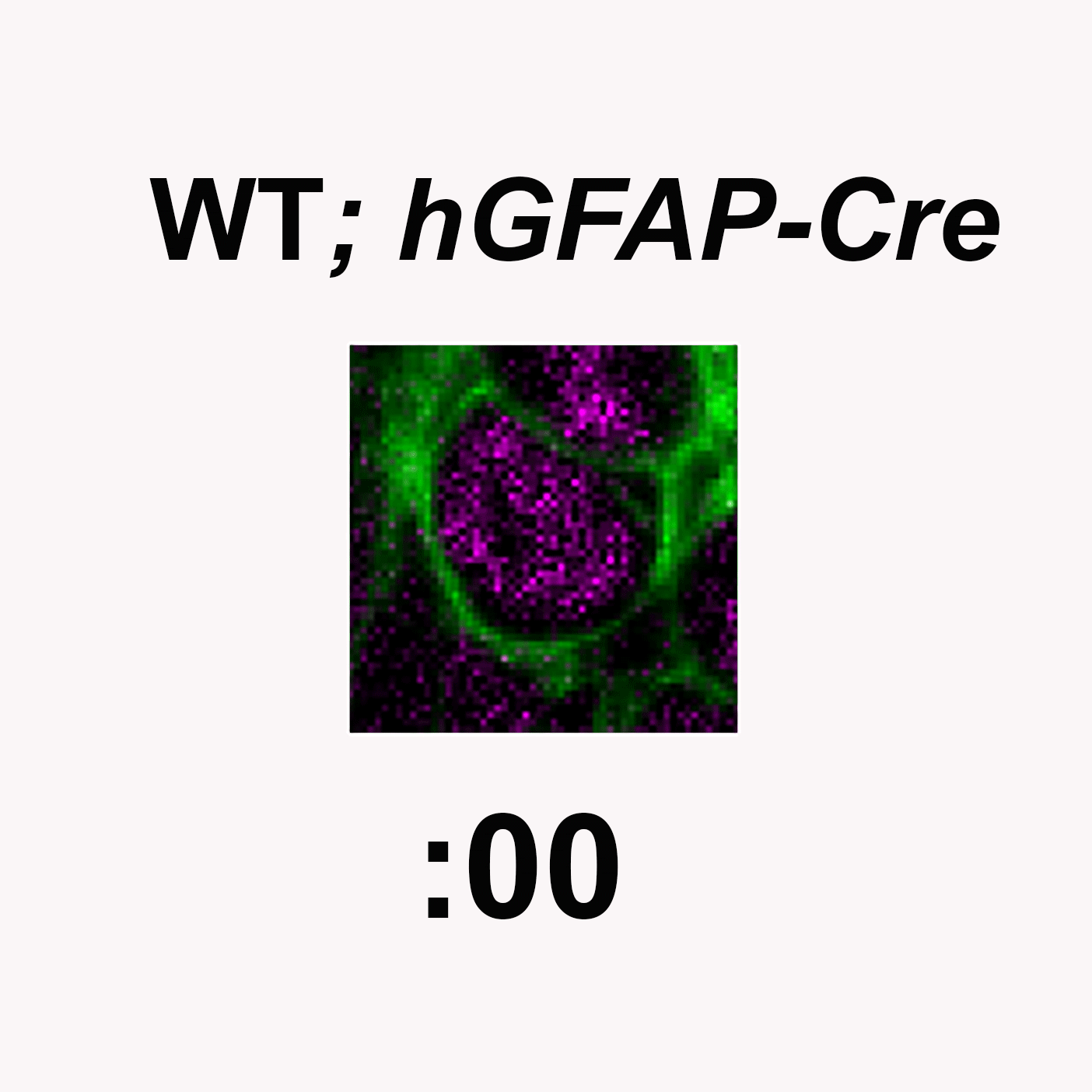

Supplement: Supplementary file 4 — Supplementary Movie. 2. WT neural progenitors undergo timely mitosis. [file 41467_2022_35719_MOESM4_ESM.gif]

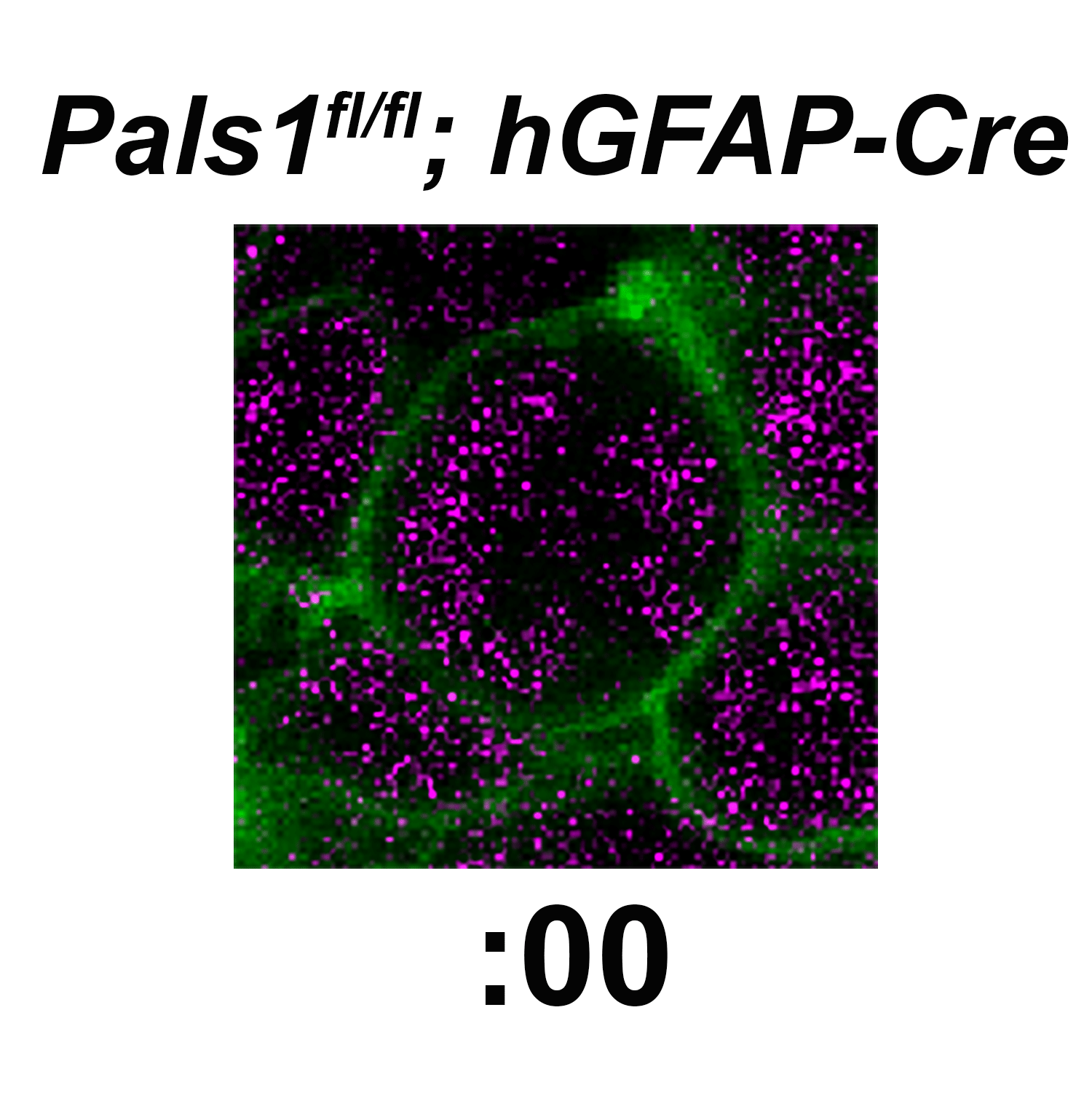

Supplement: Supplementary file 5 — Supplementary Movie. 3. PALS1-deficient neural progenitors display mitotic delay. [file 41467_2022_35719_MOESM5_ESM.gif]

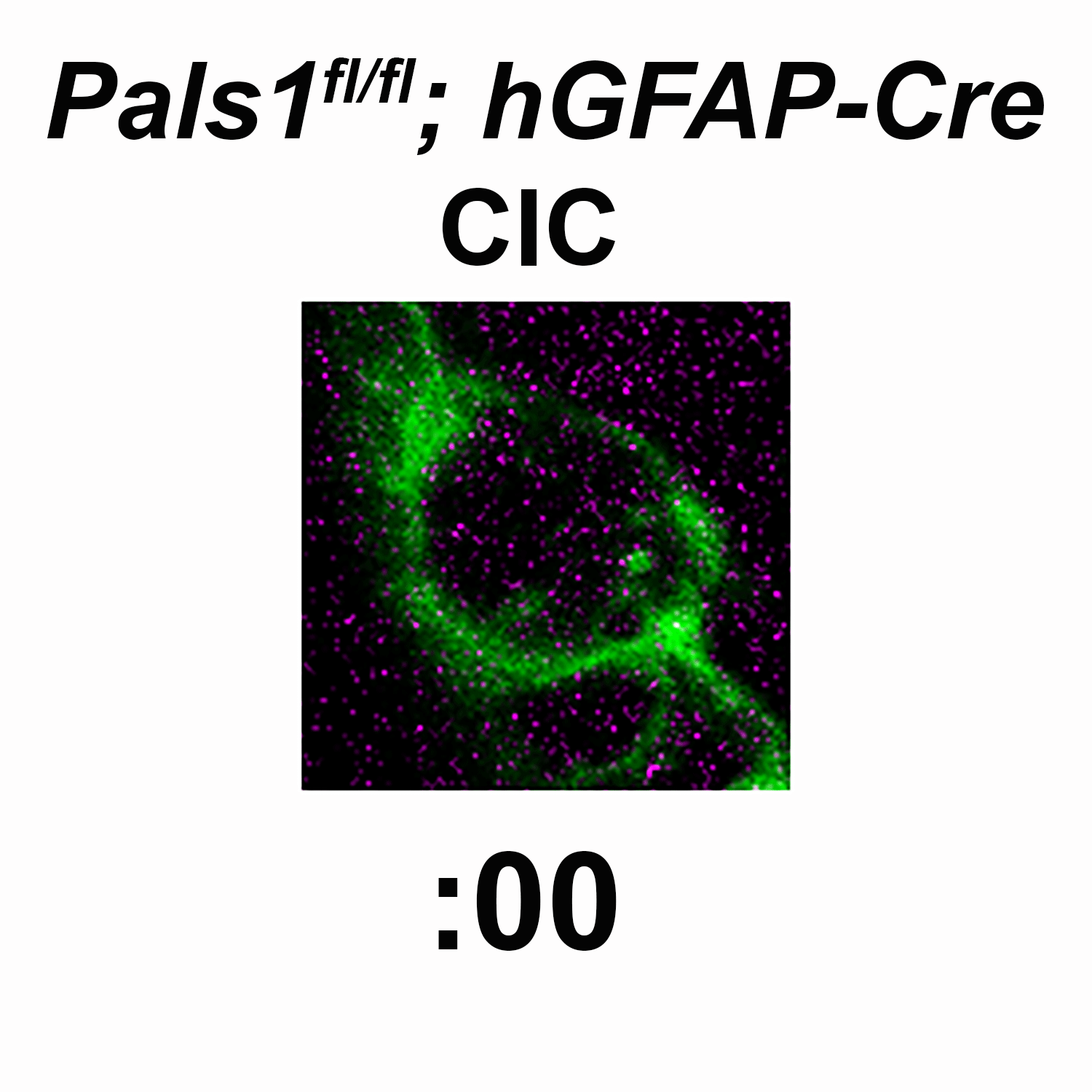

Supplement: Supplementary file 6 — Supplementary Movie. 4. PALS1-deficient neural progenitors display persistent entotic cell-in-cell structures. [file 41467_2022_35719_MOESM6_ESM.gif]

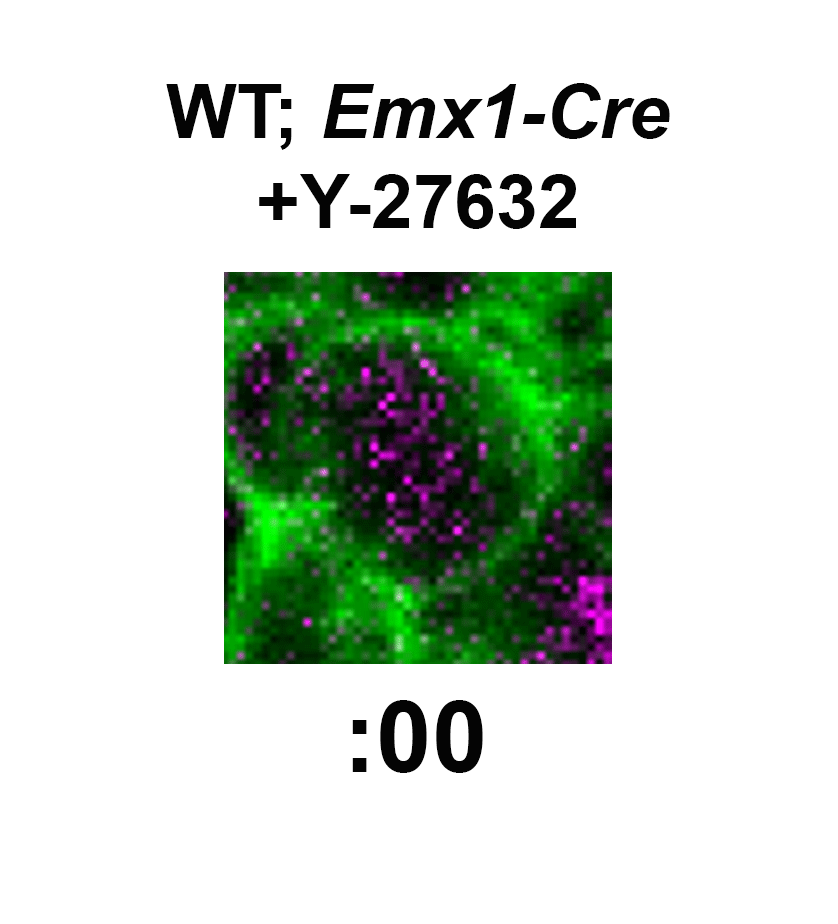

Supplement: Supplementary file 7 — Supplementary Movie. 5. Inhibition of ROCK does not prevent timely mitosis in WT neural progenitors. [file 41467_2022_35719_MOESM7_ESM.gif]

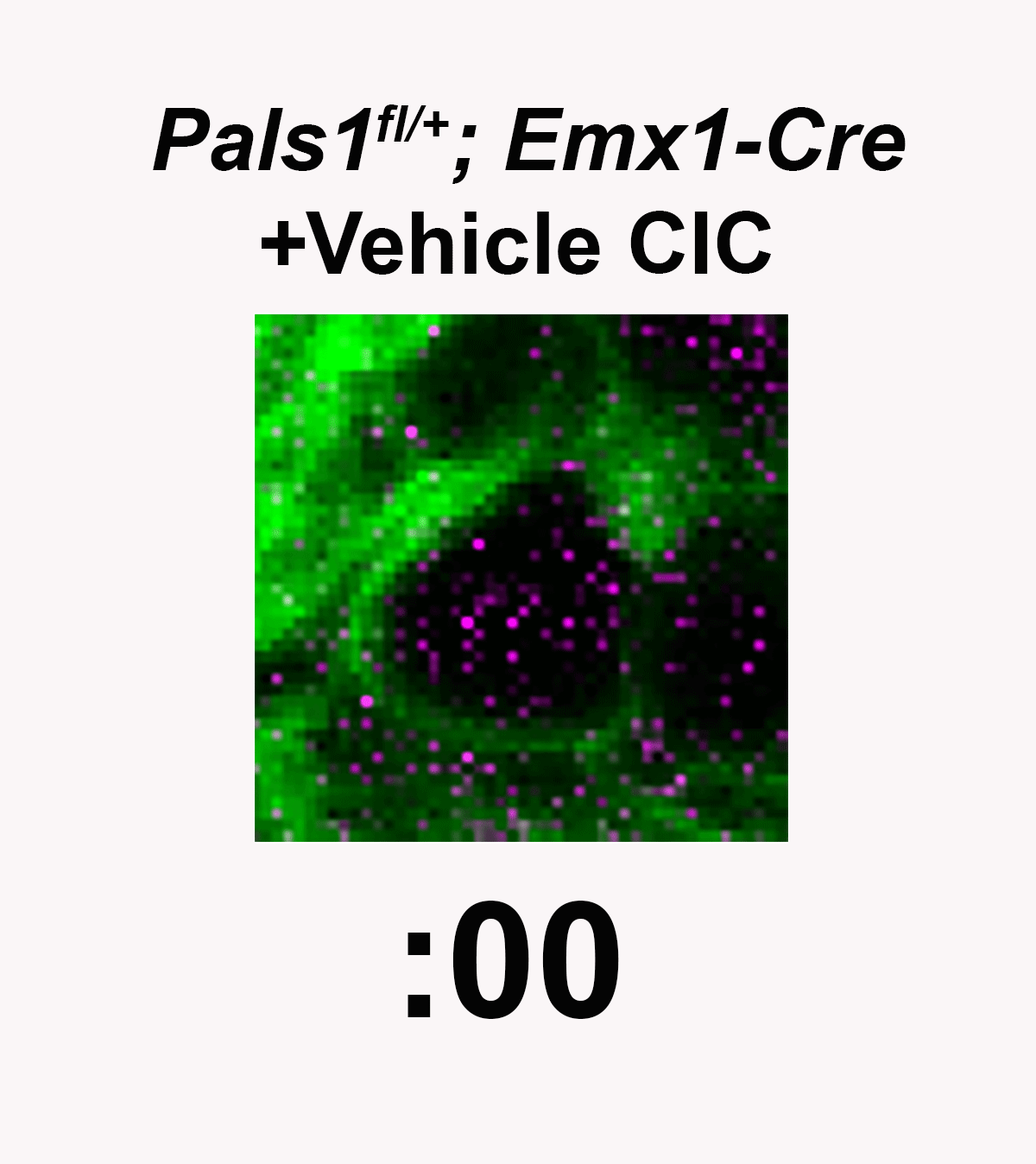

Supplement: Supplementary file 8 — Supplementary Movie. 6. PALS1-deficient neural progenitors treated with Vehicle display entotic CIC structures. [file 41467_2022_35719_MOESM8_ESM.gif]

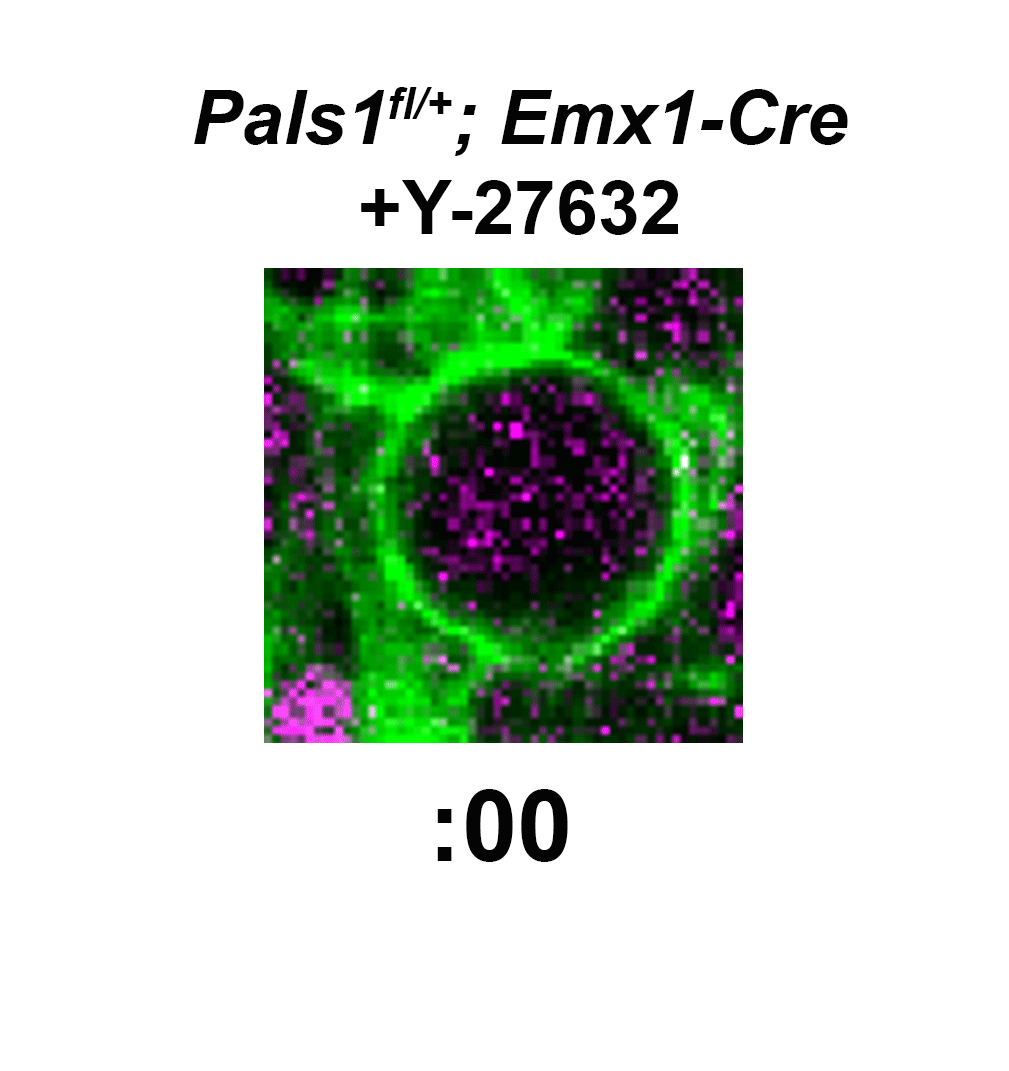

Supplement: Supplementary file 9 — Supplementary Movie. 7. ROCK inhibition allows PALS1-deficient progenitors to undergo timely mitosis. [file 41467_2022_35719_MOESM9_ESM.gif]

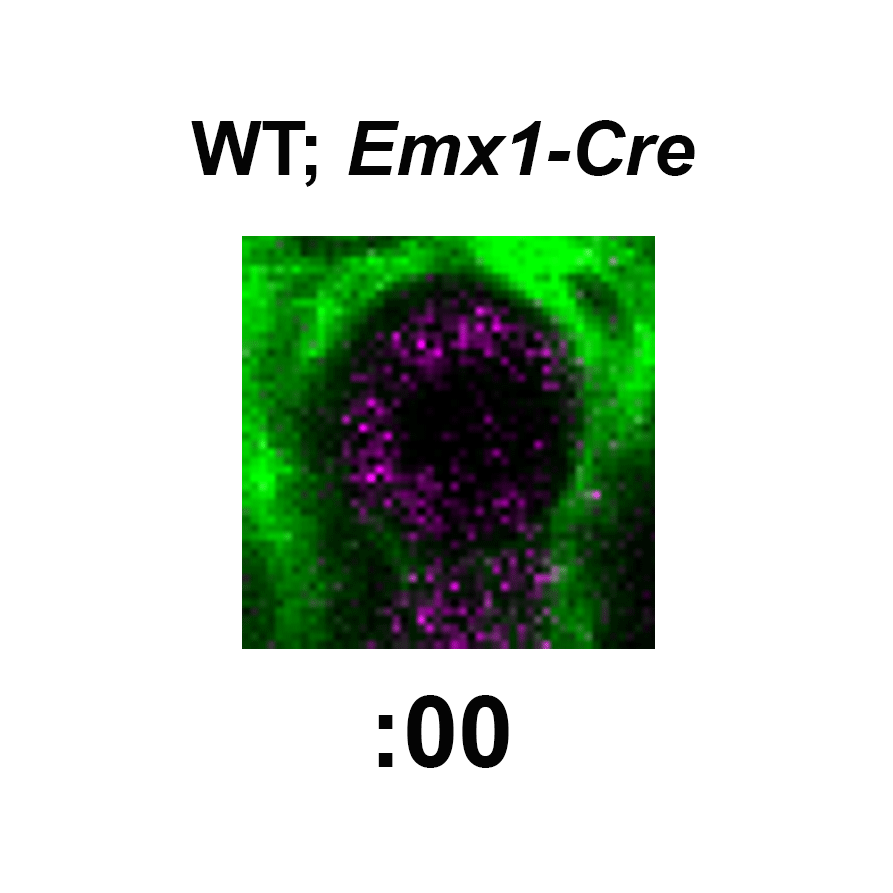

Supplement: Supplementary file 10 — Supplementary Movie. 8. Wild-type neural progenitors undergo timely mitosis. [file 41467_2022_35719_MOESM10_ESM.gif]

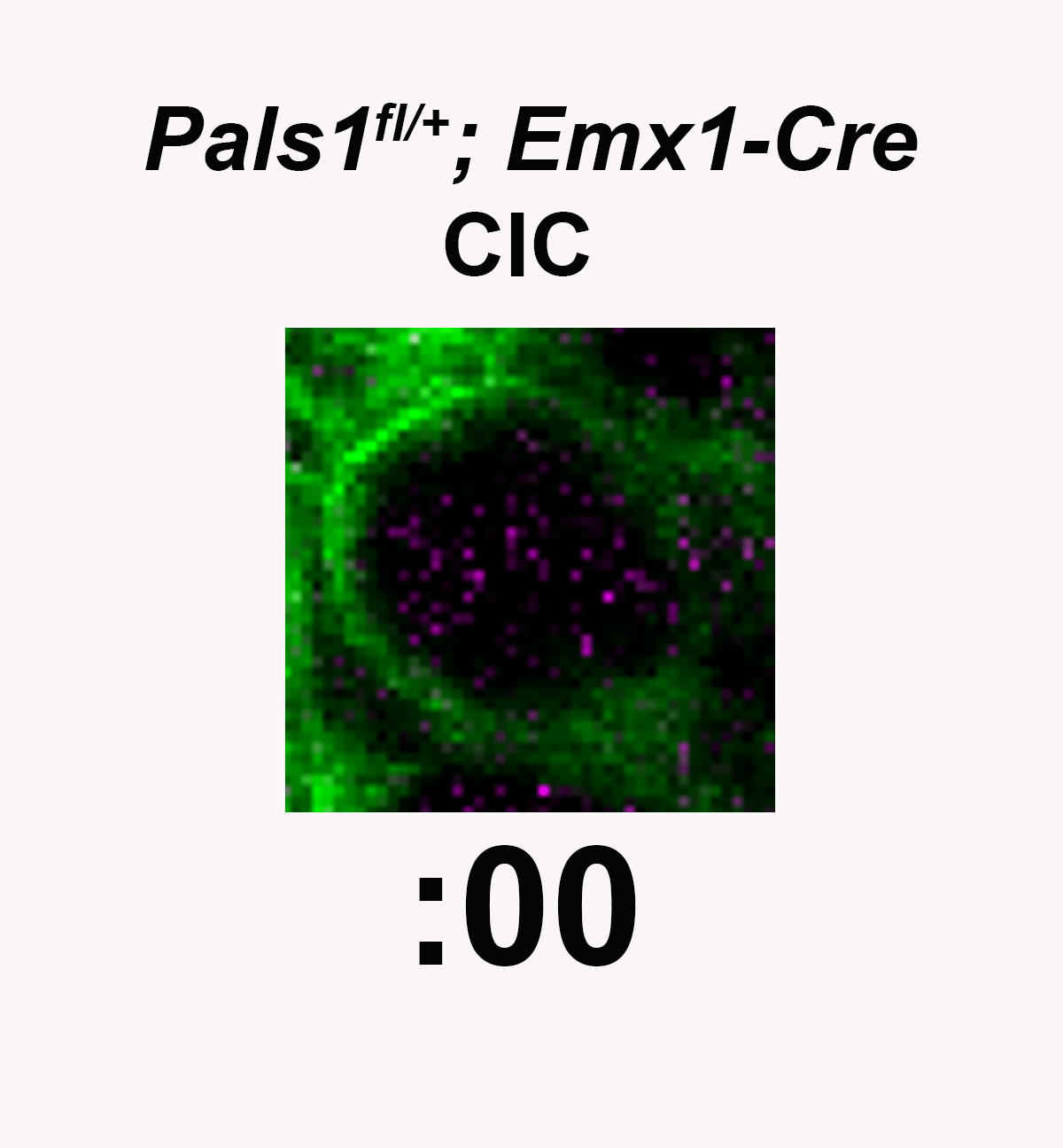

Supplement: Supplementary file 11 — Supplementary Movie. 9. PALS1-deficient neural progenitors display persistent entotic cell-in-cell structures. [file 41467_2022_35719_MOESM11_ESM.gif]

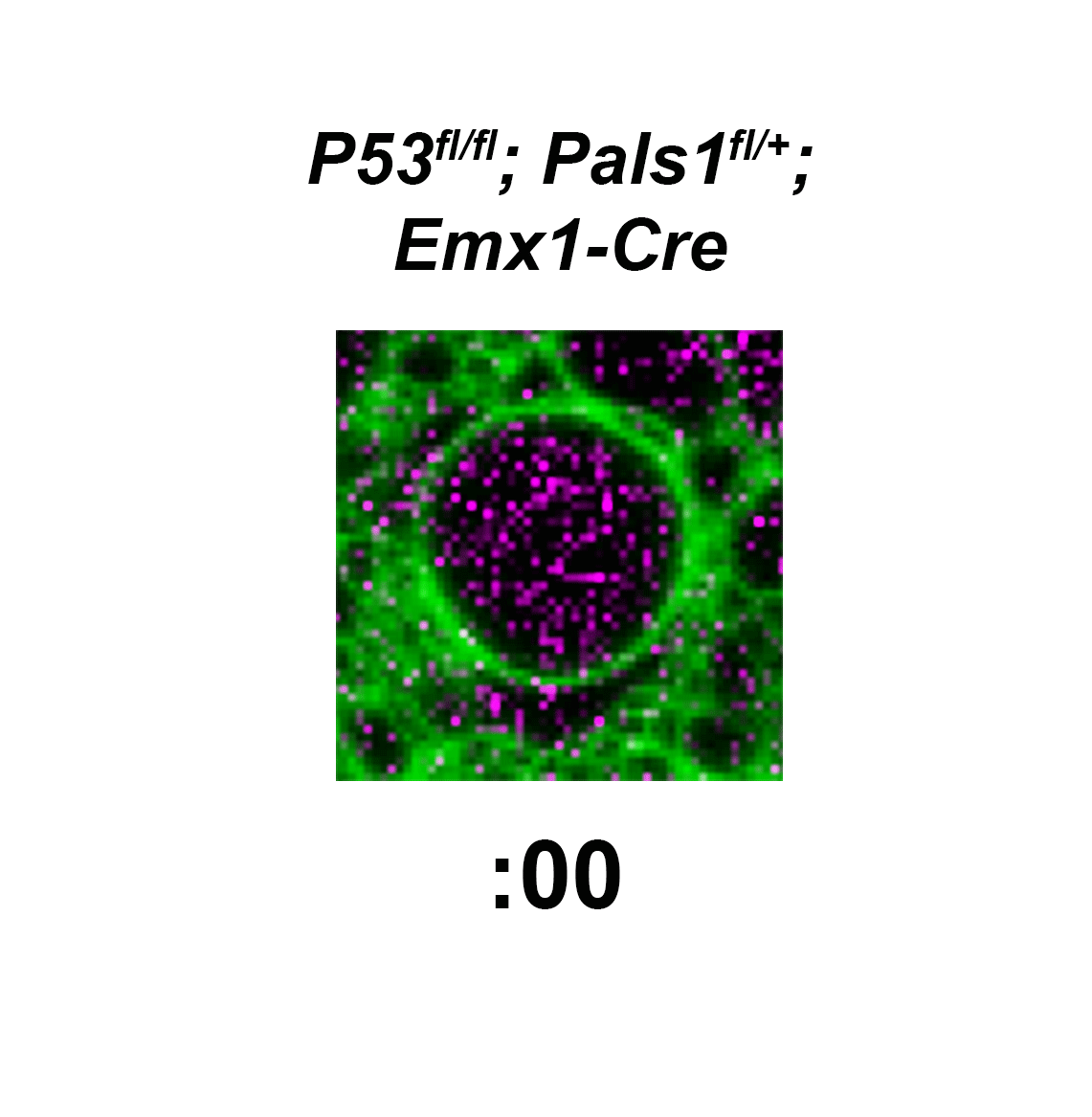

Supplement: Supplementary file 12 — Supplementary Movie. 10. Deletion of Trp53 significantly rescues mitotic defects caused by loss of PALS1. [file 41467_2022_35719_MOESM12_ESM.gif]
